# Differences in the severity and mortality risk factors for patients hospitalized for COVID-19 pneumonia between the early wave and the very late stage of the pandemic

**DOI:** 10.3389/fmed.2023.1238713

**Published:** 2023-09-28

**Authors:** Haiyan Li, Xiaoni Jia, Yu Wang, Yali Lv, Jing Wang, Yuyao Zhai, Xiaorong Xue

**Affiliations:** ^1^Department of Pharmacy, Xi’an People’s Hospital (Xi’an Fourth Hospital), Xi’an, China; ^2^Department of Science and Education, Xi’an Mental Health Center, Xi’an, China; ^3^Department of Pharmacy, Xi’an Mental Health Center, Xi’an, China; ^4^Department of Endocrinology, Xi’an People’s Hospital (Xi’an Fourth Hospital), Xi’an, China; ^5^Department of Neurology, Xi’an People’s Hospital (Xi’an Fourth Hospital), Xi’an, China

**Keywords:** coronavirus disease 2019 (COVID-19), risk factors, severity, mortality, pneumonia, Omicron

## Abstract

**Background:**

Since China’s dynamic zero-COVID policy is cancelled on December 7, 2022, the rapidly growing number of patients has brought a major public health challenge. This study aimed to assess whether there were differences in the severity and mortality risk factors for patients hospitalized for COVID-19 pneumonia between the early wave and the very late stage of the pandemic.

**Methods:**

A retrospective cross-sectional study was carried out using data from 223 hospitalized patients diagnosed with COVID-19 pneumonia during the Omicron surge in Xi’an People’s Hospital (Xi’an Fourth Hospital) from December 8, 2022, to January 31, 2023. Univariable and multivariable logistic regression analyses were used to identify potential risk factors associated with the severity and mortality of COVID-19 pneumonia during the first wave of the pandemic after the dynamic zero-COVID policy was retracted. Differences in the severity and mortality risk factors were assessed at different stages of the pandemic, mainly from demographic, clinical manifestation, laboratory tests and radiological findings of patients on admission.

**Results:**

The mean age of the 223 participants was 71.2 ± 17.4. Compared with the patients in the initial stage of the pandemic, the most common manifestation among patients in this study was cough (90.6%), rather than fever (79.4%). Different from the initial stage of the pandemic, older age, chest tightness, elevated neutrophil-to-lymphocyte ratio (NLR), decreased albumin (ALB) level and ground glass opacification (GGO) in radiological finding were identified as severity risk factors, instead of mortality risk factors for COVID-19 patients in the very late stage of the pandemic. Arterial partial pressure of oxygen/fraction of inspired oxygen (PaO_2_/FiO_2_) ≤300 mmHg, cardiovascular disease and laboratory findings including elevated levels of D-dimer, α-hydroxybutyrate dehydrogenase (α-HBDH), total bilirubin (TBIL), alanine aminotransferase (ALT), urea nitrogen (BUN), creatinine (CR), fasting blood glucose (FBG) and decreased platelet count (PLT) were still associated with mortality in the very late stage of the pandemic.

**Conclusion:**

Monitoring continuously differences in the severity and mortality risk factors for COVID-19 patients between different stages of the pandemic could provide evidence for exploring uncharted territory in the coming post-pandemic era.

## Introduction

The World Health Organization declares Coronavirus disease 2019 (COVID-19) as a global pandemic on March 11, 2020. The COVID-19 pandemic, caused by infection of severe acute respiratory syndrome coronavirus 2 (SARS-CoV-2), has led to an alarming number of infections and deaths worldwide since it is first reported in December 2019 (1). As of June 7, 2023, over 767 million confirmed cases and over 6.9 million deaths have been reported globally.

China’s strict dynamic zero-COVID policy has effectively contained the spread of COVID-19 and controlled the number of infections and death rates at a low level for close to 3 years. The number of new cases of COVID-19 has dropped rapidly due to strict prevention and control policies, and the epidemic has been effectively managed (2). Since October 2022, a new outbreak of COVID-19 has swept through nearly every province and region of China. By employing extensive testing and strict quarantine measures, it still becomes extremely difficult to protect against highly contagious infections caused by repeated waves of Omicron subvariants (3). On December 7, 2022, China’s National Health Commission announces major changes on the COVID-19 policies, which marks the end of China’s dynamic zero-COVID policy (4). Since then, a series of maintaining policies have been gradually abandoned, such as quarantine facilities, lockdowns, mass testing, and strict restrictions on mobility. Asymptomatic patients and those with mild symptoms are allowed to stay at home. Because of critical shortage of hospital beds, only patients with severe illness are admitted to the hospital. On December 26, 2022, China’s National Health Commission declares that China will manage COVID-19 with measures against Class B infectious diseases (5). These measures are implemented from January 8, 2023. In response to the growing domestic outbreaks, China continues to update the latest treatment protocols and has developed 10 versions of clinical guidelines against COVID-19. China’s National Health Commission issues the tenth edition of “Diagnosis and Treatment Protocol for Novel Coronavirus Infection (Trial)” on January 5, 2023, in which the name of the disease is revised from “novel coronavirus pneumonia” to “novel coronavirus infection” (6). This means the focus of epidemic control in China has shifted from “prevention” to “protecting health and preventing severe diseases” (7).

The vaccine is regarded as the optimal tool for protecting against infection and a protective factor for the severity and mortality of COVID-19 disease progression (8). Among the patients hospitalized for COVID-19, full vaccination is associated with reduced risk of developing severe COVID-19 (9). Patients in the initial stage of the pandemic were not vaccinated. According to the data released by Shaanxi Provincial Centre for Disease Control and Prevention, 95.3% of the individuals aged 60, and 85.4% of those over 80 in Shaanxi province, have been fully vaccinated by December 3, 2022 (10). Previous studies suggest that Omicron shows reduced clinical severity compared to the Delta variant (9). China experiences the peak of the epidemic from December 2022 to early February 2023 after the strict dynamic zero-COVID policy was retracted. On January 14, 2023, China’s National Health Commission has reported that nearly 60,000 people have died from coronavirus outbreak since December 8, 2022. The rapid increasing number of patients, especially those who develop respiratory failure and even die in short term, has brought a major public health challenge (11). However, little is known about the clinical features and outcomes of patients in the Northwestern China during the Omicron surge. To ensure timely treatment and provide empirical experience at the epidemiological level, this study aimed to characterize differences in the severity and mortality risk factors for patients hospitalized for COVID-19 pneumonia between the early wave and the very late stage of the pandemic.

## Materials and methods

### Study design and study population

Adult inpatients (age ≥ 18 years old) diagnosed with COVID-19 pneumonia in Xi’an People’s Hospital (Xi’an Fourth Hospital) from December 8, 2022, to January 31, 2023, were included in this cross-sectional study. According to literature research and clinical experience, exceptions included pregnant women and patients with incomplete electronic medical records.

In this study, the sample size was calculated by using the following formula: *n* = *z^2^p*(1*–p*)/*d^2^*, where *n* referred to the sample size, *z* referred to coefficient of confidence interval (1.96), *p* represented prevalence rate, and *d* indicated type I error level of 0.05. The severity rate of COVID-19 patients was assumed to be 15.7% based on previous studies (12). In China, the overall death rate from COVID-19 was 11% (13). Therefore, based on the above assumptions, the minimum sample size was 203 patients. Finally, 223 inpatients were included in this study.

According to the tenth edition of “Diagnosis and Treatment Protocol for Novel Coronavirus Infection” (6), the clinical types of inpatients with COVID-19 infection were as follows: 1. Mild (mild clinical symptoms with no sign of pneumonia on imaging); 2. Moderate (fever, respiratory symptoms, and imaging manifestations of pneumonia); 3. Severe (patients met one of the following criteria: respiratory distress and respiratory rate (RR) ≥30 breaths per minute; arterial oxygen saturation (SaO_2_) ≤93% at rest; arterial partial pressure of oxygen /fraction of inspired oxygen (PaO_2_/FiO_2_) ≤300 mmHg (1 mmHg = 0.133 kPa); lung infiltration >50% within 24 ~ 48 h); and 4. Critical (patients met any of the following criteria: respiratory failure occurs and mechanical ventilation is required; shock occurs; concomitant failure of other organs occurs and intensive care unit monitoring and treatment is required). The severity of the disease was evaluated within 48 h of hospital admission. To better understand the clinical features, this study classified moderate cases into the non-severe group (*n* = 150) and the severe and critical cases into the severe group (*n* = 73).

### Data collection

Data on the patients’ demographic and clinical characteristics, laboratory tests, radiological findings at admission, treatments and outcomes were extracted from electronic medical records. All data were collected by two pharmacists independently and verified by two additional clinicians. To get the laboratory results, the indicators that could reflect the blood routine, inflammatory status, cardiac function, coagulation function, hepatorenal function, and blood glucose level, were collected. Radiologic evaluation was performed using chest X-rays or chest computed tomography (CT) scans. Besides, the information concerning drug treatments accepted by inpatients, including antiviral drugs, antibiotics, corticosteroids, intravenous immunoglobulin, anticoagulant and Chinese herbs, was also collected. Further, patients would receive supplemental oxygen inhalation including nasal catheter for oxygen, face mask oxygen inhalation, high-flow oxygen, noninvasive ventilation and tracheal intubation if necessary. Two outcomes were evaluated: hospital discharge and in-hospital death. When patients’ condition got improved obviously (demonstrated by the stable vital signs, the temperature had returned to normal for more than 24 h, the acute exudative disease on the lung image was significantly improved, the patients could be converted to oral drug treatment, and there were no complications that need further treatment), the patients could get discharged from the hospital. An in-hospital death is defined as a death that occurred during hospitalization.

### Outcome measurements

The study endpoint was the risk factors associated with the severity and mortality of COVID-19 pneumonia during the first wave of the pandemic after the dynamic zero-COVID policy was retracted in Xi’an, China. Differences in the severity and mortality risk factors for patients hospitalized for COVID-19 pneumonia were assessed between the early wave and the very late stage of the pandemic.

### Statistical analysis

Descriptive statistics were presented using frequencies (percentages) for categorical variables and median (interquartile range) for abnormal continuous variables. Continuous variables of all laboratory tests were converted into categorical variables according to their reference range. Candidate variables of the patients’ demographic and clinical characteristics, laboratory tests, and radiological findings at admission were included initially. Then, 4 laboratory variables were excluded for a missing rate > 20%, including interleukin (IL)-6, brain natriuretic peptide (BNP), myoglobin, and troponin I. Differences in the candidate variables between non-severe and severe inpatients, as well as the candidate variables between the patients discharged from hospital and those died in hospital, were evaluated using the Chi-square test for categorical variables, the Mann–Whitney test for continuous variables. Univariable and multivariable ordinal logistic regression analyses were performed to identify the independent factors associated with the severity and mortality of COVID-19 pneumonia. Variables found to be significant at *p* value <0.05 from the univariable logistic regression, along with age and sex, were included in the multivariable logistic regression model. As for vital signs, part of the criterion for distinguishing the disease severity, they were not included in the regression analyses of factors associated with the severity of the disease. All statistical analyses were performed using SPSS V26.0 Statistical Software Package for Windows. A *p* value <0.05 was considered statistically significant.

## Results

### Demographic and clinical characteristics

Of these 223 patients, 150 (67.3%) patients were categorized into the non-severe group, while 73 (32.7%) patients were categorized into the severe group. A total of 174 (78.0%) patients were discharged from the hospital, while 49 (22.0%) patients died in hospital. The demographic, clinical characteristics, laboratory tests, radiological findings at admission, treatments and outcomes of 223 patients were shown in Table 1. The median age of the 223 participants was 75 (IQR, 60.0–85.0) years old, and the majority of severe (58, 79.5%) and death (44, 89.8%) cases occurred in patients aged 65 or above. There were significant differences in the age-grading between the non-severe and severe groups, as well as the discharged and the death groups (*p* < 0.05). Males accounted for 67.3%. Most of the patients (183, 82.1%) suffered from at least one of the comorbidities. Hypertension and cardiovascular disease were the most common comorbidity, with 118 (52.9%) and 100 (44.8%) patients, respectively, whilst chronic liver disease and dementia were the rarer, with 11 (4.9%) and 7 (3.1%) patients, respectively. The proportions of cardiovascular disease in the death group were higher than those in the discharged group, and the difference was significant (*p* < 0.05). In terms of clinical manifestation, the incidences of cough (202, 90.6%) in COVID-19 patients were higher than fever (177, 79.4%). The incidences of chest tightness were significantly different between the severe patients (46.6%) and the non-severe (22.0%) patients (*p* < 0.05). The incidences of consciousness disorders were significantly different between the death group (12.2%) and the discharged group (2.9%) (*p* < 0.05). Pulse velocity in the death group was significantly higher than that in the discharged group (*p* < 0.05). There were significant differences in the RR, SaO_2_ and PaO_2_/FiO_2_ between the non-severe and severe groups, as well as the discharged and the death groups (*p* < 0.05).

**Table 1:** Demographic, clinical characteristics, laboratory tests, radiological findings at admission, treatments and outcomes of 223 patients with COVID-19.

Characteristics	Total (*n* = 223)	Non-severe (*n* = 150)	Severe (*n* = 73)	Value of *p*	Discharge (*n* = 174)	Death (*n* = 49)	Value of *p*
Demographic and clinical characteristics							
Age, Median (IQR)	75.0 (60.0–85.0)	71.5 (56.0–84.0)	79.0 (67.5–86.0)	**0.007**	71.0 (57.0–84.0)	84.0 (74.5–87.5)	**<0.001**
Age (years)							
<65	68 (30.50)	53 (35.30)	15 (20.50)	**0.009**	63 (36.21)	5 (10.20)	**<0.001**
≥65	155 (69.50)	97 (64.70)	58 (79.50)		111 (63.79)	44 (89.80)	
Gender				0.378			0.720
Female	73 (32.74)	52 (34.67)	21 (28.77)		58 (33.33)	15 (30.61)	
Male	150 (67.26)	98 (65.33)	52 (71.23)		116 (66.67)	34 (69.39)	
Smoking status				0.182			0.616
Non-smoker	202 (90.58)	132 (88.00)	70 (95.89)		157 (90.23)	45 (91.84)	
Current smoker	15 (6.73)	13 (8.67)	2 (2.74)		13 (7.47)	2 (4.08)	
Ex-smoker	6 (2.69)	5 (3.33)	1 (1.37)		4 (2.30)	2 (4.08)	
Residence				0.092			**0.002**
Rural	38 (17.04)	30 (20.00)	8 (10.96)		37 (21.26)	1 (2.04)	
Urban	185 (82.96)	120 (80.00)	65 (89.04)		137 (78.74)	48 (97.96)	
Comorbidity							
Hypertension	118 (52.91)	76 (50.67)	42 (57.53)	0.335	89 (51.15)	29 (59.18)	0.320
Cardiovascular disease	100 (44.84)	64 (42.67)	36 (49.32)	0.349	68 (39.08)	32 (65.31)	**0.001**
Diabetes mellitus	62 (27.80)	37 (24.67)	25 (34.25)	0.134	43 (24.71)	19 (38.78)	0.052
Chronic pulmonary disease	53 (23.77)	35 (23.33)	18 (24.66)	0.827	46 (26.44)	7 (14.29)	0.078
Chronic renal disease	41 (18.39)	26 (17.33)	15 (20.55)	0.561	29 (16.67)	12 (24.49)	0.212
Chronic liver disease	11 (4.93)	9 (6.00)	2 (2.74)	0.468	9 (5.17)	2 (4.08)	1.000
Cancer	14 (6.28)	10 (6.67)	4 (5.48)	0.961	9 (5.17)	5 (10.20)	0.342
Dementia	7 (3.14)	5 (3.33)	2 (2.74)	1.000	4 (2.30)	3 (6.12)	0.372
Clinical manifestations							
Fever	177 (79.37)	115 (76.67)	62 (84.93)	0.152	138 (79.31)	39 (79.59)	0.966
Cough	202 (90.58)	135 (90.00)	67 (91.78)	0.669	161 (92.53)	41 (83.67)	0.110
Shortness of breath	105 (47.09)	64 (42.67)	41 (56.16)	0.058	79 (45.40)	26 (53.06)	0.343
Fatigue	76 (34.08)	54 (36.00)	22 (30.14)	0.386	64 (36.78)	12 (24.49)	0.109
Chest tightness	67 (30.04)	33 (22.00)	34 (46.58)	**<0.001**	47 (27.01)	20 (40.82)	0.063
Myalgia	34 (15.25)	24 (16.00)	10 (13.70)	0.654	29 (16.67)	5 (10.20)	0.266
Sore throat	19 (8.52)	13 (8.67)	6 (8.22)	0.911	16 (9.20)	3 (6.12)	0.696
Vomiting	16 (7.17)	11 (7.33)	5 (6.85)	0.961	10 (5.75)	4 (8.16)	0.778
Headache	13 (5.83)	8 (5.33)	5 (6.85)	0.882	10 (5.75)	3 (6.12)	1.000
Consciousness disorder	13 (5.83)	8 (5.33)	5 (6.85)	1.000	5 (2.87)	6 (12.24)	**0.021**
Chest pain	10 (4.48)	6 (4.00)	4 (5.48)	1.000	7 (4.02)	1 (2.04)	0.823
Vital signs, Median (IQR)							
Pulse (bpm)^a^	84 (76.0–94.0)	82 (75.7–91.0)	86 (76.0–96.5)	0.595	81.5 (75.0–90.0)	91 (77.0–100.0)	**0.012**
RR (bpm)^b^	20 (19.0–20.0)	20 (19.0–20.0)	20 (19.0–22.0)	**0.002**	20.0 (19.0–20.0)	20.0 (19.0–22.0)	**0.001**
SaO_2_ (%)	94.6 (90.8–97.2)	96.8 (95.1–98.2)	88.9 (85.4–91.6)	**<0.001**	273 (236.0–335.0)	88.7 (85.6–93.9)	**0.002**
PaO_2_/FiO_2_ (mmHg)	336 (279.5–425.0)	413 (351.0–479.0)	268.5 (240.5–294.5)	**<0.001**	357.5 (303.5–432.25)	95.3 (92.0–97.3)	**0.020**
Laboratory and radiological data							
Blood routine							
WBC (⨯10^9^/L)	5.9 (4.4–8.6)	5.7 (4.1–8.0)	6.9 (4.7–10.0)	**0.040**	5.6 (4.4–8.1)	7.6 (4.5–9.3)	**0.016**
N (⨯10^9^/L)	4.3 (3.0–7.2)	4.0 (2.7–5.9)	5.9 (3.8–9.6)	**<0.001**	4.0 (3.0–6.1)	6.6 (3.3–8.2)	**0.001**
N% (%)	76.3 (66.9–86.3)	73.0 (64.4–82.1)	84.5 (74.9–90.5)	**<0.001**	74.5 (66.5–83.2)	86.0 (75.4–91.5)	**<0.001**
L(⨯10^9^/L)	0.8 (0.5–1.1)	0.9 (0.6–1.3)	0.6 (0.4–0.8)	**<0.001**	0.9 (0.6–1.2)	0.6 (0.3–0.8)	**<0.001**
L% (%)	14.3 (7.7–22.9)	16.9 (10.8–25.7)	8.3 (5.1–14.3)	**<0.001**	23.5 (9.0–15.6)	8.0 (5.5–12.8)	**<0.001**
NLR	5.3 (3.0–11.5)	4.3 (2.6–7.3)	10.4 (5.2–19.9)	**<0.001**	4.7 (2.9–9.6)	11.0 (6.3–21.9)	**<0.001**
HB (g/L)	124.0 (110.0–137.0)	125.0 (109.8–137.3)	120.0 (110.0–134.0)	0.539	125.5 (111.0–135.0)	120.5 (104.0–137.0)	0.291
PLT (⨯10^9^/L)	175.0 (131.0–237.0)	175.0 (131.3–232.5)	178.0 (130.0–245.0)	0.929	178.0 (132.0–244.0)	153.0 (104.0–213.0)	**0.008**
Inflammatory markers							
CRP (mg/L)	48.12 (12.72–106.59)	30.6 (9.0–71.8)	94.2 (54.6–160.2)	**<0.001**	39.2 (9.0–85.7)	106.1 (66.8–158.0)	**<0.001**
PCT (ng/mL)	0.10 (0.04–0.44)	0.06 (0.04–0.20)	0.20 (0.08–1.12)	**<0.001**	0.07 (0.04–0.20)	0.48 (0.10–1.80)	**<0.001**
Coagulation indicators							
PT (s)	13.2 (12.5–14.0)	13.2 (12.5–13.9)	13.3 (12.7–14.3)	0.271	13.2 (12.5–13.9)	13.5 (12.6–14.7)	0.066
APTT (s)	35.8 (30.4–40.1)	35.8 (30.9–39.6)	35.9 (29.6–42.6)	0.836	35.4 (29.7–39.3)	36.1 (29.6–42.8)	0.139
D-dimer (mg/L)	0.9 (0.4–1.8)	0.6 (0.3–1.4)	1.4 (0.7–2.4)	**<0.001**	0.7 (0.4–1.3)	1.9 (1.0–3.8)	**<0.001**
Cardiac function							
CK (U/L)	86.0 (49.9–168.6)	72.0 (45.8.1–137.4)	116.9 (57.7–250.5)	**0.006**	72.2 (49.8–158.0)	123.1 (56.0–338.0)	**0.010**
LDH (U/L)	237.5 (189.8–323.0)	213.0 (174.8–274.3)	305.5 (239.0–413.0)	**<0.001**	215.0 (181.0–275.0)	343.0 (248.0–575.0)	**<0.001**
α-HBDH (U/L)	186.9 (148.7–242.0)	165.5 (138.4–219.9)	238.3 (205.5–314.9)	**<0.001**	167.3 (140.8–218.9)	276.4 (218.6–405.7)	**<0.001**
Hepatorenal function							
TBIL (μmol/L)	12.4 (9.1–17.9)	12.0 (9.0–16.3)	13.6 (9.5–21.1)	0.093	12.2 (9.2–16.3)	13.9 (10.4–24.1)	0.070
AST (U/L)	25.0 (19.0–38.3)	25.0 (19.0–36.3)	30.0 (20.3–50.0)	**0.029**	24.0 (19.0–35.0)	37.0 (22.0–55.0)	**<0.001**
ALT (U/L)	19.0 (4.40–8.58)	19.0 (13.0–30.0)	23.0 (13.0–35.5)	0.209	17.5 (13.0–29.0)	26.0 (16.0–36.0)	**0.014**
ALB (g/L)	34.0 (13.0–31.0)	34.9 (31.2–38.0)	31.6 (29.0–35.0)	**<0.001**	34.2 (31.2–37.8)	31.0 (27.1–34.6)	**<0.001**
BUN (mmol/L)	5.9 (4.1–9.4)	5.2 (3.9–8.3)	6.9 (4.8–12.6)	**0.003**	5.3 (4.0–8.0)	9.4 (6.2–14.8)	**<0.001**
CR (μmol/L)	70.1 (58.4–105.5)	68.2 (58.1–101.2)	73.8 (61.1–123.3)	0.117	69.9 (58.2–96.4)	100.5 (63.5–140.2)	**0.022**
FBG (mmol/L)	6.5 (5.3–8.8)	6.2 (4.9–7.9)	7.6 (6.0–9.9)	**<0.001**	6.4 (5.2–8.2)	8.5 (6.5–12.1)	**<0.001**
Radiological features							
DPS	132 (59.19)	96 (64.00)	36 (49.32)	**0.036**	108 (62.07)	24 (48.98)	0.100
GGO	74 (33.18)	40 (26.67)	34 (46.58)	**0.003**	60 (34.48)	14 (28.57)	0.438
Consolidation	14 (6.28)	6 (4.00)	8 (10.96)	0.086	10 (5.75)	4 (8.16)	0.778
Fibrosis	47 (21.08)	27 (18.00)	20 (27.40)	0.156	34 (19.54)	13 (26.53)	0.296
Pleural effusion	25 (11.21)	13 (8.67)	12 (16.44)	0.084	19 (10.92)	6 (12.24)	0.795
Lesion range				0.073			0.124
Unilateral lung	28 (12.56)	23 (15.33)	5 (6.85)		25 (14.37)	3 (6.12)	
Bilateral lung	195 (87.44)	127 (84.67)	68 (93.15)		149 (85.63)	46 (93.88)	
Medicinal treatment							
Antiviral drugs							
Paxlovid	73 (32.74)	45 (30.00)	28 (38.36)	0.212	59 (33.91)	14 (28.57)	0.482
Azvudine	56 (25.11)	31 (20.67)	25 (34.25)	**0.028**	30 (17.24)	26 (53.06)	**<0.001**
Paxlovid + Azvudine	12 (5.38)	9 (6.00)	3 (4.11)	0.787	9 (5.17)	3 (6.12)	1.000
Antibiotic	210 (94.17)	139 (92.67)	71 (97.26)	0.285	162 (93.10)	48 (97.96)	0.349
Glucocorticoids	126 (56.50)	75 (50.00)	51 (69.86)	**0.005**	92 (52.87)	34 (69.39)	**0.039**
Immunoglobulin	18 (8.07)	9 (6.00)	9 (12.33)	0.104	12 (6.90)	6 (12.24)	0.359
Anticoagulants	71 (31.84)	40 (26.67)	31 (42.47)	**0.017**	54 (31.03)	17 (34.69)	0.627
Chinese herbs	13 (5.83)	11 (7.33)	2 (2.74)	0.285	12 (6.90)	1 (2.04)	0.349
Oxygen mode				**<0.001**			**<0.001**
NO	12 (5.38)	10 (6.67)	2 (2.74)		11 (6.32)	1 (2.04)	
NC/FM	192 (86.10)	133 (88.67)	59 (80.82)		158 (90.80)	34 (69.39)	
HF/NIV	6 (2.69)	5 (3.33)	1 (1.37)		2 (1.15)	4 (8.16)	
TI	13 (5.83)	2 (1.33)	11 (15.07)		3 (1.72)	10 (20.41)	

Data are presented as medians (interquartile ranges, IQR) and N (%). ^a^bpm, beats per minute; ^b^bpm, breaths per minute; RR, respiratory rate; SaO_2_, arterial oxygen saturation; PaO_2_/FiO_2_, arterial partial pressure of oxygen/fraction of inspired oxygen; WBC, white blood cell count; N, neutrophil count; L, lymphocyte count; NLR, neutrophil-to-lymphocyte ratio; HB, hemoglobin; PLT, platelet count; CRP, C-reactive protein; PCT, procalcitonin; PT, prothrombin time; APTT, activated partial thromboplastin time; CK, creatine kinase; LDH, lactate dehydrogenase; α,HBDH, α,hydroxybutyrate dehydrogenase; TBIL, total bilirubin; AST, aspartate aminotransferase; ALT, alanine aminotransferase; ALB, albumin; BUN, urea nitrogen; CR, creatinine; FBG, fasting blood glucose; DPS, diffuse plaques shadow; GGO, ground glass opacification; Paxlovid, Nirmatrelvir Tablets/Ritonavir Tablets (co-packaged); NO, no oxygen inhalation; NC/FM, nasal catheter for oxygen/face mask oxygen inhalation; HF/NIV, high-flow oxygen/noninvasive ventilation; TI, tracheal intubation.

### Laboratory and radiological findings

The following parameters had statistical difference between the non-severe and severe groups: white blood cell count (WBC), neutrophil count (N), N%, lymphocyte count (L), L%, neutrophil-to-lymphocyte ratio (NLR), C-reactive protein (CRP), procalcitonin (PCT), D-dimer, creatine kinase (CK), lactate dehydrogenase (LDH), α-Hydroxybutyrate dehydrogenase (α-HBDH), aspartate aminotransferase (AST), albumin (ALB), urea nitrogen (BUN) and fasting blood glucose (FBG). Between the discharged and death groups, the following parameters had statistical difference: WBC, N, N%, L, L%, NLR, platelet count (PLT), CRP, PCT, D-dimer, CK, LDH, α-HBDH, AST, alanine aminotransferase (ALT), ALB, BUN, creatinine (CR) and FBG.

Diffuse plaques shadow (DPS) (59.2%) and ground glass opacification (GGO) (33.2%) were typical manifestations of radiological findings in COVID-19 patients. GGO was significantly more frequently observed in the severe group than the non-severe group (*p* < 0.05), while DPS was significantly more frequently observed in the non-severe group than the severe group (*p* < 0.05).

### Treatments

Effective SARS-CoV-2 antivirals would alleviate severe cases and reduce mortality. As shown in Table 1, a total of 129 patients (57.8%) received antiviral treatments, including nirmatrelvir/ritonavir and azvudine, indicating widespread use of antivirals in patients with COVID-19 during the first wave of the pandemic after the dynamic zero-COVID policy was retracted. Antibiotics were used by 94.2%, glucocorticoids by 56.5% and anticoagulants by 31.8% of the patients. Compared with the non-severe group, azvudine, glucocorticoids and anticoagulants treatment were more frequently administered in the severe group (*p* < 0.05). Compared with the discharged group, azvudine and glucocorticoids treatment were more frequently administered in the death group (*p* < 0.05). In addition, oxygen therapy was administered in 94.6% of the inpatients, and there were significant differences in the oxygen mode between the non-severe group and severe group, as well as the discharged group and the death group (*p* < 0.001).

### Risk factors for the severity of disease in 223 patients with COVID-19

Univariable and multivariable logistic regression analyses of demographic and clinical factors associated with the severity of COVID-19 were shown in Table 2, and laboratory and radiological factors associated with the severity of COVID-19 were shown in Table 3. In the univariable analyses, nineteen factors were significantly associated with increasing risks of the severity of COVID-19 pneumonia: age ≥ 65 years, chest tightness, WBC ≤10⨯10^9^/L, N > 7⨯10^9^/L, N% >70, L < 0.8⨯10^9^/L, L% <20, NLR >4.4, PLT <100⨯10^9^/L, CRP >10 mg/L, PCT >0.25 ng/mL, D-dimer >0.55 mg/L, CK >190 U/L, LDH >220 U/L, α-HBDH >182 U/L, ALB <35 g/L, FBG >6.1 mmol/L, DPS and GGO in chest imaging examination.

**Table 2:** Univariable and multivariable logistic regression analyses of demographic and clinical factors associated with the severity of COVID-19.

Variable	Unadjusted OR (95% CI)	Value of *p*	Adjusted OR (95% CI)	Value of *p*
Demographic characteristics				
Age (≥65 vs. <65), years	2.113 (1.093–4.084)	**0.026**	2.171 (1.096–4.297)	**0.029**
Gender (male vs. female)	1.314 (0.715–2.414)	0.379	1.326 (0.701–2.510)	0.385
Smoking status	0.290 (0.064–1.322)^a^	0.110		
	0.377 (0.043–3.292)^b^	0.378		
Residence (urban vs. rural)	2.031 (0.880–4.688)	0.097		
Comorbidity				
Hypertension	1.319 (0.751–2.318)	0.336		
Cardiovascular disease	1.307 (0.746–2.292)	0.349		
Diabetes mellitus	1.591 (0.865–2.926)	0.136		
Chronic pulmonary disease	1.075 (0.560–2.066)	0.827		
Chronic renal disease	1.233 (0.608–2.503)	0.561		
Chronic liver disease	0.441 (0.093–2.097)	0.304		
Cancer	0.812 (0.246–2.681)	0.732		
Dementia	0.817 (0.155–4.314)	0.812		
Clinical manifestations				
Fever	1.715 (0.815–3.612)	0.155		
Cough	1.241 (0.461–3.343)	0.670		
Shortness of breath	1.722 (0.979–3.027)	0.059		
Fatigue	0.767 (0.420–1.399)	0.387		
Chest tightness	3.091 (1.695–5.635)	**<0.001**	3.095 (1.682–5.694)	**<0.001**
Myalgia	0.833 (0.375–1.850)	0.654		
Sore throat	0.944 (0.344–2.592)	0.911		
Vomiting	0.812 (0.246–2.681)	0.732		
Headache	1.305 (0.412–4.139)	0.651		
Consciousness disorders	1.184 (0.335–4.182)	0.793		
Chest pain	1.243 (0.289–5.349)	0.770		

Bold values indicated a value of *p* < 0.05. OR, odds ratio; CI, confidence interval.

^a^Current smoker vs. non-smoker, ^b^ex-smoker vs. non-smoker.

**Table 3:** Univariable and multivariable logistic regression analyses of laboratory and radiological factors associated with the severity of COVID-19.

Variable	Unadjusted OR (95% CI)	Value of *p*	Adjusted OR (95% CI)	Value of *p*
Blood routine				
WBC (⨯10^9^/L) (≤10 vs. >10)	0.443 (0.216–0.908)	**0.026**		
N (⨯10^9^/L) (>7 vs. ≤7)	3.362 (1.802–6.273)	**<0.001**		
N% (%) (>70 vs. ≤70)	5.268 (2.357–11.772)	**<0.001**		
L (⨯10^9^/L) (<0.8 vs. ≥0.8)	4.156 (2.269–7.613)	**<0.001**		
L% (%) (<20 vs. ≥20)	4.688 (2.234–9.841)	**<0.001**		
NLR (>4.4 vs. ≤4.4)	4.740 (2.402–9.354)	**<0.001**	2.683 (1.172–6.141)	**0.020**
HB (g/L) (<110 vs. ≥110)	0.927 (0.480–1.789)	0.822		
PLT (⨯10^9^/L) (<100 vs. ≥100)	2.484 (1.003–6.153)	**0.049**		
Inflammatory markers				
CRP (mg/L) (>10 vs. ≤10)	7.464 (2.568–21.694)	**<0.001**		
PCT (ng/mL) (>0.25 vs. ≤0.25)	2.761 (1.496–5.098)	**0.001**		
Coagulation indicators				
PT (s) (>12.1 vs. ≤12.1)	1.745 (0.669–4.552)	0.255		
APTT (s) (>40 vs. ≤40)	1.405 (0.757–2.606)	0.281		
D-dimer (mg/L) (>0.55 vs. ≤0.55)	4.314 (2.097–8.877)	**<0.001**		
Cardiac function				
CK (U/L) (>190 vs. ≤190)	2.563 (1.253–5.242)	**0.010**		
LDH (U/L) (>220 vs. ≤220)	6.518 (3.064–13.866)	**<0.001**		
α-HBDH (U/L) (>182 vs. ≤182)	6.434 (3.185–12.998)	**<0.001**	5.465 (2.556–11.684)	**<0.001**
Hepatorenal function				
TBIL (μmol/L) (>20.5 vs. ≤20.5)	1.879 (0.933–3.784)	0.077		
AST (U/L) (>34 vs. ≤34)	1.678 (0.931–3.025)	0.085		
ALT (U/L) (>55 vs. ≤55)	1.212 (0.456–3.222)	0.701		
ALB (g/L) (<35 vs. ≥35)	2.714 (1.465–5.027)	**0.001**	2.270 (1.053–4.896)	**0.037**
BUN (mmol/L) (>7.4 vs. ≤7.4.)	1.736 (0.973–3.098)	0.062		
CR (μmol/L) (>110.5 vs. ≤110.5)	1.704 (0.889–3.264)	0.108		
FBG (mmol/L) (>6.1 vs. ≤6.1)	2.487 (1.347–4.592)	**0.004**		
Radiological features				
DPS	0.547 (0.310–0.965)	**0.037**		
GGO	2.397 (1.335–4.304)	**0.003**	2.417 (1.158–5.047)	**0.010**
Consolidation	2.954 (0.985–8.859)	0.053		
Fibrosis	1.636 (0.826–3.242)	0.158		
Pleural effusion	2.073 (0.894–4.805)	0.089		
Lesion range (Bilateral vs. Unilateral lung)	2.463 (0.896–6.769)	0.081		

Bold values indicated a value of *p* < 0.05.

Multivariable logistic regression analyses revealed that aged 65 years or above (adjusted odds ratio [OR] and 95% confidence interval [CI], 2.171 [1.096, 4.297]; *p* = 0.029), chest tightness (adjusted OR 3.095 [1.682, 5.694]; *p* < 0.001), NLR >4.4 (adjusted OR 2.683 [1.172, 6.141]; *p* = 0.020), α-HBDH >182 U/L (adjusted OR 5.465 [2.556, 11.684]; *p* < 0.001), albumin <35 g/L (adjusted OR 2.270 [1.053, 4.896]; *p* = 0.037), and GGO (adjusted OR 2.417 [1.158, 5.047]; *p* = 0.010) in radiological finding were independent risk factors associated with the severity of COVID-19 pneumonia.

### Risk factors for in-hospital death of disease in 223 patients with COVID-19

Univariable and multivariable logistic regression analyses of demographic and clinical factors associated with in-hospital death of COVID-19 were shown in Table 4, and laboratory and radiological factors associated with in-hospital death of COVID-19 were shown in Table 5. In the univariable analyses, 28 factors were significantly associated with increasing risks of the mortality of COVID-19 pneumonia: aged 65 years or above, live in urban areas, cardiovascular disease, consciousness disorders, RR ≥30 breaths per minute, SaO_2_ ≤ 93%, PaO_2_/FiO_2_ ≤ 300 mmHg, WBC ≤10⨯10^9^/L, N > 7⨯10^9^/L, N% >70, L < 0.8⨯10^9^/L, L% <20, NLR >4.4, PLT <100⨯10^9^/L, CRP >10 mg/L, PCT >0.25 ng/mL, APTT >40 s, D-dimer >0.55 mg/L, CK >190 U/L, LDH >220 U/L, α-HBDH >182 U/L, total bilirubin (TBIL) >20.5 μmol/L, AST >34 U/L, ALT >55 U/L, ALB <35 g/L, BUN >7.4 mmol/L, CR >110.5 μmol/L and FBG >6.1 mmol/L.

**Table 4:** Univariable and multivariable logistic regression analyses of demographic and clinical factors associated with in-hospital death of COVID-19.

Variable	Unadjusted OR (95% CI)	Value of *p*	Adjusted OR (95% CI)	Value of *p*
Demographic characteristics				
Age (≥65 vs. <65)- years	4.995 (1.883–13.245)	**0.001**		
Gender (male vs. female)	1.133 (0.572–2.247)	0.720		
Smoking status	0.537 (0.117–2.467)^a^	0.424		
	1.744 (0.309–9.834)^b^	0.528		
Residence (urban vs. rural)	0.077 (0.010–0.578)	**0.013**		
Comorbidity				
Hypertension	1.385 (0.728–2.633)	0.321		
Cardiovascular disease	2.934 (1.513–5.691)	**0.001**	2.747 (1.214–6.220)	**0.015**
Diabetes mellitus	1.929 (0.987–3.771)	0.055		
Chronic pulmonary disease	0.464 (0.195–1.105)	0.083		
Chronic renal disease	1.622 (0.756–3.480)	0.215		
Chronic liver disease	0.780 (0.163–3.735)	0.756		
Cancer	2.083 (0.665–6.532)	0.208		
Dementia	2.772 (0.599–12.826)	0.192		
Clinical manifestations				
Fever	1.017 (0.464–2.232)	0.966		
Cough	0.414 (0.161–1.065)	0.067		
Shortness of breath	1.359 (0.720–2.566)	0.344		
Fatigue	0.557 (0.271–1.146)	0.112		
Chest tightness	1.864 (0.963–3.608)	0.065		
Myalgia	0.568 (0.208–1.556)	0.271		
Sore throat	0.644 (0.180–2.307)	0.499		
Vomiting	1.458 (0.437–4.867)	0.540		
Headache	1.070 (0.283–4.048)	0.921		
Consciousness disorders	4.716 (1.374–16.186)	**0.014**		
Chest pain	0.497 (0.060–4.140)	0.518		
Vital signs				
Pulse (bpm) (>100 vs. ≤100)	1.772 (0.775–4.047)	0.175		
RR (bpm) (≥30 vs. <30)	9.773 (1.834–52.066)	**0.008**		
SaO_2_ (%) (≤93% vs. >93%)	4.559 (2.137–9.726)	**<0.001**		
PaO_2_/FiO_2_ (mmHg) (≤300 vs. >300)	5.333 (2.486–11.442)	**<0.001**	4.716 (2.115–10.518)	**<0.001**

Bold values indicated a value of *p* < 0.05.

**Table 5:** Univariable and multivariable logistic regression analyses of laboratory and radiological factors associated with in-hospital death of COVID-19.

Variable	Unadjusted OR (95% CI)	Value of *p*	Adjusted OR (95% CI)	Value of *p*
Blood routine				
WBC (⨯10^9^/L) (≤10 vs. >10)	0.381 (0.178–0.814)	**0.013**		
N (⨯10^9^/L) (>7 vs. ≤7)	3.123 (1.595–6.116)	**0.001**		
N% (%) (>70 vs. ≤70)	2.222 (1.010–4.891)	**0.021**		
L (⨯10^9^/L) (<0.8 vs. ≥0.8)	4.473 (2.182–9.170)	**<0.001**		
L% (%) (<20 vs. ≥20)	3.132 (1.383–7.090)	**0.006**		
NLR (>4.4 vs. ≤4.4)	5.348 (2.277–12.559)	**<0.001**		
HB (g/L) (<110 vs. ≥110)	1.967 (0.985–3.927)	0.055		
PLT (⨯10^9^/L) (<100 vs. ≥100)	3.800 (1.507–9.581)	**0.005**	15.149 (3.255–70.508)	**0.001**
Inflammatory markers				
CRP (mg/L) (>10 vs. ≤10)	3.955 (1.346–11.618)	**0.012**		
PCT (ng/mL) (>0.25 vs. ≤0.25)	5.746 (2.879–11.469)	**<0.001**		
Coagulation indicators				
PT (s) (>12.1 vs. ≤12.1)	1.224 (0.436–3.433)	0.701		
APTT (s) (>40 vs. ≤40)	3.800 (1.507–9.581)	**0.036**		
D-dimer (mg/L) (>0.55 vs. ≤0.55)	17.380 (4.085–73.943)	**<0.001**	9.483 (1.773–50.728)	**0.009**
Cardiac function				
CK (U/L) (>190 vs. ≤190)	2.294 (1.070–4.921)	**0.033**		
LDH (U/L) (>220 vs. ≤220)	10.385 (3.546–30.412)	**<0.001**		
α-HBDH (U/L) (>182 vs. ≤182)	11.323 (4.226–30.338)	**<0.001**	8.709 (2.787–27.217)	**0.001**
Hepatorenal function				
TBIL (μmol/L) (>20.5 vs. ≤20.5)	2.929 (1.402–6.121)	**0.004**	4.588 (1.479–14.225)	**0.008**
AST (U/L) (>34 vs. ≤34)	2.793 (1.454–5.363)	**0.002**		
ALT (U/L) (>55 vs. ≤55)	2.820 (1.066–7.464)	**0.037**	5.438 (1.022–28.920)	**0.047**
ALB (g/L) (<35 vs. ≥35)	3.590 (1.683–7.656)	**0.001**		
BUN (mmol/L) (>7.4 vs. ≤7.4)	3.963 (2.042–7.693)	**<0.001**	4.320 (1.676–11.137)	**0.002**
CR (μmol/L) (>110.5 vs. ≤110.5)	4.190 (2.095–8.383)	**<0.001**	6.430 (2.155–19.186)	**0.001**
FBG (mmol/L) (>6.1 vs. ≤6.1)	3.783 (1.730–8.269)	**0.001**	5.892 (1.646–21.084)	**0.006**
Radiological features				
DPS	0.587 (0.310–1.111)	0.102		
GGO	0.760 (0.380–1.522)	0.438		
Consolidation	1.458 (0.437–4.867)	0.540		
Fibrosis	1.496 (0.701–3.193)	0.298		
Pleural effusion	1.138 (0.428–3.027)	0.795		
Lesion range (Bilateral vs. Unilateral lung)	0.389 (0.112–1.346)	0.136		

Bold values indicated a value of *p* < 0.05.

Multivariable logistic regression analyses revealed that cardiovascular disease (adjusted OR 2.747 [1.214, 6.220]; *p* = 0.015), PaO_2_/FiO_2_ ≤ 300 mmHg (adjusted OR 4.716 [2.115, 10.518]; *p* < 0.001), PLT <100⨯10^9^/L (adjusted OR 15.149 [3.255, 70.508]; *p* = 0.001), D-dimer>0.55 mg/L (adjusted OR 9.483 [1.773, 50.728]; *p* = 0.009), α-HBDH>182 U/L (adjusted OR 8.709 [2.787, 27.217]; *p* = 0.001), TBIL >20.5 μmol/L (adjusted OR 4.588 [1.479, 14.225]; *p* = 0.008), ALT >55 U/L (adjusted OR 5.438 [1.022, 28.920]; *p* = 0.047), BUN >7.4 mmol/L (adjusted OR 4.320 [1.676, 11.137]; *p* = 0.002), CR >110.5 μmol/L (adjusted OR 6.430 [2.155, 19.186]; *p* = 0.001), and FBG >6.1 mmol/L (adjusted OR 5.892 [1.646, 21.084]; *p* = 0.006) were independent risk factors associated with the mortality of COVID-19 pneumonia.

## Discussion

Cough and fever remained to be the dominant symptoms in the very late stage of the pandemic. Different from the situation where the incidences of fever in COVID-19 patients were higher than cough during the initial phase of the pandemic (2, 14–23), this study showed that the incidences of cough in COVID-19 patients were higher than that of fever in the very late stage of the pandemic. In addition, this study also found that the proportion of severe patients (15.1%) without fever in the very late stage was higher than the proportion (6.6%) in the initial stage (15). The positive association of high fever and acute respiratory distress syndrome (ARDS) was found at the early stage of COVID-19 (14). This phenomenon indicated that the severity of clinical manifestation of COVID-19 got mitigated significantly during the Omicron predominant period, compared with the first phase of the pandemic. This study showed the symptom of chest tightness was associated with the severity of COVID-19. The symptom of chest tightness was reported as a characteristic of COVID-19 patients who experienced exacerbations (24). Another study showed that chest tightness was a risk factor for mortality of severe COVID-19 patients (18), which was inconsistent with this study. Clinicians should monitor closely the patients with the symptom of chest tightness and adjust treatment regimens to prevent the deterioration of the disease. Several studies had identified cardiovascular disease as an independent predictor of mortality in COVID-19 patients (22, 23, 25), which was consistent with this study. More attention should be paid to patients with cardiovascular disease to prevent the progression and deterioration of COVID-19. PaO_2_/FiO_2_ ≤ 300 was an independent risk factor of disease mortality in adult COVID-19 patients in this study. It is reported that PaO_2_/FiO_2_ < 200 mmHg on admission is associated with poor prognosis in COVID-19 patients (26). Another study showed that PaO_2_/FiO_2_ was an independent risk factor of mortality for intensive care COVID-19 patients (27), which was consistent with this study. These results suggested that those patients with these features on admission should be monitored closely to achieve better outcomes.

In this study, the median age of severely ill patients was higher than that in previous studies (15–18, 23). Based on robust studies, increasing age was an uncontested risk factor for disease severity (2, 15, 23, 28, 29). Older age may influence pathogenesis, not only in terms of the likelihood of increasing prevalence of comorbidities with age, but also the lower immune response (14, 30). In fact, the immune system becomes less effective over time and then further affect the quality and quantity of immune system cells (30). Literature has demonstrated that individuals aged 65 or above have the hazard rate of ARDS 3.26 times than those under 65 (14). This study revealed that people aged 65 or above was an important predictor of disease severity during the Omicron surge, which was consistent with previously published studies (16). It was also reported older age was associated with greater risk of death from COVID-19 infection during the initial stage of the pandemic (14, 18, 21–23, 25, 30–32), which was inconsistent with this study. The findings in this study accorded with the results of previous study, regarding older age as a risk factor for poor survival only in the first wave (33).

Laboratory biomarkers provided a useful tool for the severity and mortality prediction of COVID-19 patients. Laboratory indicators including NLR >4.4, α-HBDH >182 U/L and albumin <35 g/L, were identified as independent predictors of disease severity, while PLT <100⨯10^9^/L, D-dimer >0.55 mg/L, α-HBDH >182 U/L, TBIL >20.5 μmol/L, ALT >55 U/L, BUN >7.4 mmol/L, CR >110.5 μmol/L and FBG >6.1 mmol/L were identified as independent predictors of in-hospital death in this study. Elevated NLR levels reflected enhancing inflammatory processes and could indicate a poor prognosis (34). This study had shown that elevated NLR was an independent risk factor associated with COVID-19 severity, which was consistent with previously published studies (17, 28, 34–37). Literature also demonstrates that NLR is an independent risk factor for mortality in hospitalized patients with COVID-19 (38). Patients with elevated NLR should be given more attention to avoid further deterioration or even death. Decreased albumin was demonstrated the predictor of disease severity in COVID-19 pneumonia (17), which was consistent with this study. Literature has also demonstrated that decreasing albumin levels are associated with poor outcomes and mortality in COVID-19 patients (39). Alpha-hydroxybutyrate dehydrogenase (α-HBDH) is an auxiliary marker of myocardial injury (40–41). It was identified as an independent risk factor for disease severity and mortality among COVID-19 patients in previous studies (29, 40–42), which was consistent with this study. Early monitoring of α-HBDH levels may be critical for identifying high-risk individuals in patients with COVID-19. COVID-19 progression and mortality are closely associated with multiple organ damage (20). Indicators of impaired liver and kidney function are closely associated with the progression of COVID-19. It is reported that the elevated levels of AST and TBIL on admission are independently associated with increasing risks of mortality (22, 39, 43), but the association between the elevated level of ALT and increasing risks of mortality is not so strong (39, 44). The elevated level of ALT was identified as an independent factor associated with COVID-19 mortality in this study, which was consistent with the study by Wang et al. (27) showing ALT should be considered as predictor of mortality in COVID-19 patients (45). More studies are needed to validate the association between transaminitis and the risk of mortality in COVID-19 patients in the future. It was reported that elevated levels of BUN, CR and blood glucose were significantly associated with increasing risks of COVID-19 disease exacerbation or in-hospital deaths (18–19, 24, 29, 31–32, 46), which was consistent with this study. D-dimer levels in the blood indicate the activation of coagulation systems and fibrinolysis. The values of D-dimer may be helpful in predicting the evolution of COVID-19 disease. Literature has demonstrated that elevated D-dimer levels on admission are independent risk factors for death (14, 21, 31), which was consistent with this study. The findings were consistent with previous findings that decreased platelet count was associated with increased odds of in-hospital deaths (18, 31, 32). Monitoring platelets of patients during hospitalization may be important in predicting the prognosis of COVID-19 patients (47). Studies had documented that the presence of GGO in radiological findings was associated with progression to critical illness or in-hospital mortality (28, 48). Different from the initial stage of the pandemic, GGO in radiological finding was identified as severity risk factors, instead of mortality risk factor for COVID-19 patients in the very late stage of the pandemic.

Long COVID is characterized by a diverse range of pulmonary, liver, kidney, cardiovascular, neurological and gastrointestinal abnormalities (49–53). The incidences of new-onset in-hospital and persistent disorders such as diabetes, hypertension, gastrointestinal and cardiac symptoms have been reported among COVID-19 patients (49, 54–57). Of 161 patients without type-2 diabetes complications before hospitalization in our study, 43 patients (26.7%) showed fasting glucose levels higher than 7 mmol/L during their hospitalization, which was highly suggestive of diabetes. New-onset in-hospital type-2 diabetes mellitus was diagnosed in 22.6% of patients with COVID-19 in New York, which was similar to our study (54).

It was reported that there were shifts in demographics toward younger age and proportionally more females with COVID-19 across the pandemic in countries outside of China (58–60). Different from foreign countries that had been opening up for a long time, China’s strict dynamic zero-COVID policy was implemented for close to 3 years. Because of critical shortage of hospital beds, only patients with severe illness are admitted to the hospital after the strict dynamic zero-COVID policy was retracted. In addition, 24.2% of patients admitted to the hospital were vaccinated in South Africa during the Omicron wave (59), which was much lower than China (10). Considering different epidemic prevention policies and vaccination status, it is difficult to determine the evolution differences of COVID-19 patient characteristics across the pandemic between China and foreign countries. Furthermore, socioeconomic, demographic, and other population characteristics were associated with changes in population mobility in response to the COVID-19 pandemic in China and other countries (61–62).

### Strengths and limitations

This study was the first to characterize differences in the severity and mortality risk factors for patients hospitalized for COVID-19 pneumonia between the early wave and the very late stage of the pandemic. The investigation of these differences could help healthcare providers monitor susceptible population at an early stage and offer theoretical assistance for future management of this disease. This study had several limitations. Firstly, this study was conducted in a single center, which wasn’t representative of the general situation in China. Further larger and more representative studies are needed to explore how these factors affect disease severity and mortality. Secondly, candidate predictors were collected from the electronic medical records in this retrospective study. Examinations and tests were carried out based on individual specific condition. Missing data of some variables from hospitalized patients, such as detailed information of blood pressure and laboratory data including interleukin (IL)-6, brain natriuretic peptide (BNP), myoglobin, and troponin I, made it impossible to characterize differences in new-onset disorders (such as diabetes, hypertension, gastrointestinal and cardiac symptoms) for COVID-19 patients between different stages of the pandemic. Early identification of risk factors for new-onset disorders could help prevent long-term complications. However, long-term follow-up for liver, cardiac, neurological, pulmonary and endocrine/genitourinary systems complications were not conducted in our study. Thirdly, patients were categorized as non-severe and severe groups within 48 h of hospital admission based on initial clinical presentation. Patients experienced clinical deterioration (admitted as moderate cases but developed into severe cases) during hospitalization were not discriminated in our study, which might cause bias. Finally, effective treatment with antivirals for COVID-19 has been recommend in the Chinese guidelines (6). Nirmatrelvir/ritonavir and azvudine (the first homegrown anti-COVID-19 drug by China) are available during the Omicron predominant period (10), which certainly play a major role in improving patients’ survival (63, 64). Due to the diverse treatment schemes for COVID-19 among different patients and clinical departments (some patients had already received antiviral treatment before admission), the effect of treatments was not considered as candidate predictor of disease mortality.

## Conclusion and implications

Our study demonstrated that the clinical manifestations, the severity and mortality risk factors of COVID-19 between the early wave and the very late stage of the pandemic might differ. Compared with the patients in the initial stage of the pandemic, the most common manifestation among patients in the very late stage of the epidemic was cough, rather than fever. Different from the initial stage of the pandemic, older age, chest tightness, elevated NLR, decreased albumin level and GGO in radiological findings were identified as severity risk factors, instead of mortality risk factors for COVID-19 patients in the very late stage of the pandemic. PaO_2_/FiO2 ≤ 300 mmHg, cardiovascular disease and laboratory findings including elevated levels of D-dimer, α-HBDH, ALT, TBIL, BUN, CR, FBG and decreased platelet count were still associated with mortality in the very late stage of the pandemic. Monitoring continuously differences in the severity and mortality risk factors for COVID-19 patients between different stages of the pandemic could provide evidence for exploring uncharted territory in the coming post-pandemic era.

## Data availability statement

The original contributions presented in the study are included in the article/supplementary material, further inquiries can be directed to the corresponding authors.

## Ethics statement

The studies involving humans were approved by the Ethical Committee of Xi’an People’s Hospital (Xi’an Fourth Hospital) (No: 2023063). The studies were conducted in accordance with the local legislation and institutional requirements. The ethics committee/institutional review board waived the requirement of written informed consent for participation from the participants or the participants’ legal guardians/next of kin because patient informed consent was waived due to the retrospective study design.

## Author contributions

HL and YL: conceptualization. HL, YZ, XX, YW, and JW: data collection. HL and XJ: analyze the data, software, and original draft. HL, XJ, and YZ: methodology. XX and YL: supervision. YZ and YL: critical revision of the manuscript. All authors have approved the final version of the manuscript.

## Glossary

RR: respiratory rate
SaO_2_: arterial oxygen saturation
PaO_2_/FiO_2_: arterial partial pressure of oxygen/fraction of inspired oxygen
WBC: white blood cell count
N: neutrophil count
L: lymphocyte count
NLR: neutrophil-to-lymphocyte ratio
HB: hemoglobin
PLT: platelet count
CRP: C-reactive protein
PCT: procalcitonin
PT: prothrombin time
APTT: activated partial thromboplastin time
CK: creatine kinase
LDH: lactate dehydrogenase
α-HBDH: α-hydroxybutyrate dehydrogenase
TBIL: total bilirubin
AST: aspartate aminotransferase
ALT: alanine aminotransferase
ALB: albumin
BUN: urea nitrogen
CR: creatinine
FBG: fasting blood glucose
DPS: diffuse plaques shadow
GGO: ground glass opacification
Paxlovid: Nirmatrelvir Tablets/Ritonavir Tablets (co-packaged)
NO: no oxygen inhalation
NC/FM: nasal catheter for oxygen/face mask oxygen inhalation
HF/NIV: high-flow oxygen/noninvasive ventilation
TI: tracheal intubation
